# Breast cancer treatment and outcomes at Cape Coast Teaching Hospital, Ghana

**DOI:** 10.4314/gmj.v55i3.3

**Published:** 2021-09

**Authors:** Fejiro O Okifo, Derek A Tuoyire, Anthony B Appiah, Samuel Y Debrah, Martin T Morna, Rosemary B Duda

**Affiliations:** 1 Harvard Medical School, Boston, MA; 2 Department of Community Medicine, School of Medical Sciences, College of Health and Allied Sciences, University of Cape Coast, Ghana; 3 Ghana Field Epidemiology and Laboratory Training Program (GFELTP), School of Public Health, University of Ghana-Legon, Accra, Ghana; 4 Department of Surgery, School of Medical Sciences, College of Health and Allied Sciences, University of Cape Coast, Ghana; 5 Department of Surgery, Division of Surgical Oncology, Beth Israel Deaconess Hospital, Harvard Medical School, Boston, MA

**Keywords:** Breast cancer, advanced stage presentation, treatment, Cape Coast, Ghana, Sub-Saharan Africa

## Abstract

**Objectives:**

This study sought to determine the presentation, treatment and outcomes of breast cancer among women in Cape Coast, Ghana.

**Design:**

Retrospective medical record review

**Setting:**

Cape Coast Teaching Hospital, Cape Coast, Ghana

**Participants:**

Female breast cancer patients

**Interventions:**

None

**Main outcome measures:**

Proportion of female breast cancer patients presenting with advanced disease.

**Results:**

Approximately 84% of women had a primary presentation of breast cancer, with metastatic disease present in 34% of patients. Surgical management mainly involved partial mastectomy (21.7%) and total mastectomy (78.6%), with the most common postoperative complications being surgical site infections (3.8%). Non-surgical management involved chemotherapy, radiation therapy and anti-estrogen therapy, with Stage 3 and 4 patients twofold more likely to receive neoadjuvant chemotherapy than earlier stages (OR= 2.0 95% CI (1.4, 3.0, p<0.001). Grade 1 cancers were diagnosed in 11.0%, Grade 2 in 43.8%, and Grade 3 in 45.2%. The mean cancer size was 6.5 centimetres (range 1.5 to 20.0). Lymphatic vascular invasion was present in 59/125 (47.2%), estrogen receptor status was positive in 32.6%, progesterone receptors were positive in 22.1%, and Her-2/neu was positive in 32.6%. Triple-negative breast cancer was identified in 41/89 (46.1%).

**Conclusions:**

Women with breast cancer typically present to the Cape Coast Teaching Hospital with advanced stage disease and experience poor outcomes.

**Funding:**

Funding for this study was provided by the Harvard Medical School Scholars in Medicine.

## Introduction

The increasing burden of cancers in many low-and middle-income countries has become a great public health concern. Breast cancer is recognized as an increasingly important burden of disease, particularly among women in African countries. By region, Western Africa had 45,157 new patients with breast cancer in 2018 and 20,983 deaths relating to breast cancer.^1^ Adeloye et al. estimated the incidence of breast cancer in Africa as 24.5 per 100,000 person years, which represents an increase from 20.1 per 100,000 person years in 2000 to 28.9 per 100,000 person years in 2015.^2^

In Ghana, data obtained via Globocan indicates that new patients of breast cancer in 2018 represented 20.4% of all cancers in both sexes and 33.6% in females.^1^ Breast cancer had the highest incidence of new cancers, followed by cervical cancer and liver cancer.

Despite the observed increases in the incidence of breast cancer in low-and middle-income countries, the rates are still generally low compared to high-income countries.

In contrast, higher breast cancer mortality is observed in low-and middle-income countries compared with high-income countries. The difference in incidence between these countries is linked with many factors, including demographic, biological, cultural, and societal influences. For instance, studies attribute lower incidence of breast cancer among Africa women compared with their Western counterparts to an older age of menarche, increased parity, early childbearing, and extended postpartum lactation^3^–^5^African women have menarche at older ages, increased parity, childbearing at earlier ages, and extended postpartum lactation.^3^–^5^ These gynaecologic and reproductive patterns observed for African women invariably reduce their exposure to activities of reproductive hormones, which could be protective against breast cancer. On the other hand, the high breast cancer mortality among African women has been linked with multiple factors, including delayed presentation, limited therapeutic modalities, and a potential predisposition to biologically aggressive tumours. In particular, studies have shown that women with an African ancestry tend to have higher rates of triple-negative breast cancer (TNBC). Indeed while studying breast cancers among Ghanaian, African American and White American women, Stark et al.^6^ found a higher prevalence of TNBC among Ghanaian (82%) women compared with their African American (26%) and white American (16%) counterparts. TNBC breast cancers typically lack expression of the estrogen and progesterone receptors and the cell surface receptor Her2/neu. Such tumours are associated with an inherently aggressive basal breast cancer subtype as defined by gene-expression studies.^6^

The complex nature of breast cancer with its histologic subtypes and tumour behaviour patterns based on racial/ethnic variations has over the years inspired research across various communities or populations with the view to characterizing the disease in each specific population to determine suitable control measures.^7^ In Ghana, such prior efforts have largely involved studies in Accra^8^–^10^ and Kumasi,^11^, the two largest cities. To date, no such studies have been reported outside these two major cosmopolitan areas, which barely represent what pertains to other parts of the country. The current study sought to determine the presentation, treatment, and outcomes of breast cancer among women in Cape Coast, Ghana, to bridge this void. The evidence from this study should provide the initial glimpses of breast cancer in the peripheral regions of Ghana for policy intervention purposes.

## Methods

### Study setting and design

We conducted a retrospective review of medical records of breast cancer patients diagnosed from 2011 to 2019 at the Cape Coast Teaching Hospital (CCTH), Ghana. The CCTH is the largest medical facility in the central region of Ghana. With a capacity of over 400 beds, the hospital serves as the main referral centre for the satellite health facilities within the central region and some parts of the western region.^12^

### Data variables

Documents reviewed included the medical chart, operative records, pathology, and radiology reports. Data collection included the patient characteristics, gender, age at diagnosis, menopause status, family history, marital status, and town or village of residence. The operative procedure and postoperative complications and radiographic imaging were recorded. Histologic and pathologic features included histologic type, grade, size, stage of disease, and nodal status. We documented HER2-neu protein status and hormone-receptor status, such as oestrogen and progesterone receptors. Non-operative management including chemotherapy, anti-oestrogen hormonal therapy, and radiation therapy administration. Therapies were recorded as dichotomous variables.

### Statistical analysis

Data were entered into an excel spreadsheet and transferred to the IBM SPSS Statistics for Windows (Professional Analysis Software platform version 26.0, Armonk, NY). Statistical analysis included descriptive statistics for frequency and mean values, Fisher's Exact Test (FET) (1-sided), chi-square test, Pearson correlation, binary logistic regression analysis and multinomial regression analysis. A p-value < 0.05 was considered statistically significant. Correlations were performed using the continuous variable age to determine any significant association with the stage of disease, tumour markers, and tumour grade. Binary logistic regression analysis was performed to assess statistically significant associations between operative intervention, chemotherapy and radiation therapy administration and stage of the disease.

### Ethical considerations

The study protocol was reviewed and approved by the Institutional Review Board of Harvard Medical School, Boston, MA, USA (Ref.#: IRB19-0351), and the Ethical Review Committee of Cape Coast Teaching Hospital, Cape Coast, Ghana (Ref.#: CCTHERC/EC/2019/071) before the commencement of the study. Upon administrative approval from the hospital authorities, anonymized patients' records were retrieved and reviewed. No informed consent was required. The data obtained was password protected and available only to the investigators to ensure confidentiality and privacy.

## Results

### Patient characteristics

**Figure 1:**
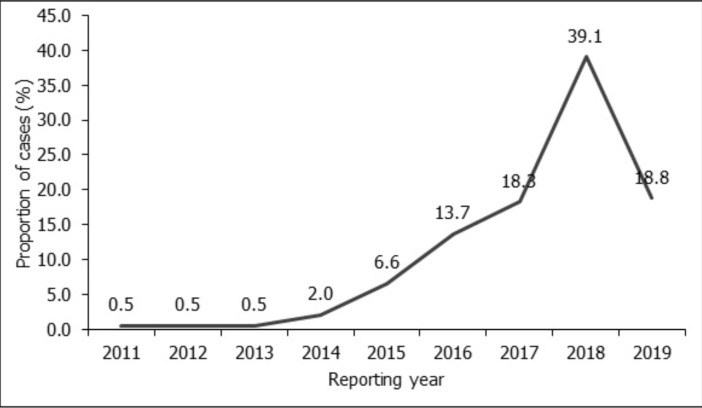
Trend of breast cancer diagnosed at Cape Coast Teaching Hospital

Records were available and reviewed for 197 patients diagnosed with breast cancer at Cape Coast Regional Hospital between September 2011 and June 2019. All patients diagnosed with breast cancer in this time interval were women. As shown in Table 1, the mean age of diagnosis was 49.9 + 12.9 years (range 22 – 87 years). As graphically presented in Figure 1, breast cancer patients diagnosed at Cape Coast Teaching Hospital began to rise remarkably from 2014 (2%) and peaked in 2018 (39%).

**Table 1:** Characteristics of breast cancer patients

Characteristics	Number(%)
**Age (n=197)**	
**≤50 years**	108 (54.8)
**>50 years**	89 (45.2)
**Menopause Status (n=156)**	
**Pre-menopausal**	53 (34.0)
**Peri and Post-menopausal**	103 (66.0)
**Presentation (n=197)**	
**Primary breast ca**	166 (84.3)
**Recurrent breast ca**	30 (15.2)
**Metastatic breast ca**	68 (34.5)
**Family History (n=197)**	
**Positive FH**	12 (6.1)
**Negative FH**	185 (93.9)
**Degree of relations (n=12)**	
**First degree relative**	6 (3.0)
**Second-degree relative**	7 (3.6)
**Staging (n=189)**	
**Stage 0**	1 (0.5)
**Stage 1**	5 (2.6)
**Stage 2**	34 (18.0)
**Stage 3**	77 (40.7)
**Stage 4**	72 (38.1)

Most women were 50 years of age or younger (54.8%) at the time of diagnosis. Menopause status was recorded for 156 women; most women were peri-menopausal or postmenopausal at the time of diagnosis (66%).

For 84.3% of women, this was the primary presentation of breast cancer. The stage of disease was recorded for 189 patients, as shown in Table 1.

Metastatic disease was documented at presentation in 34.5%. The majority of women presented with Stage 3 or Stage 4 disease (78.8%). Family history of breast cancer was uncommon. Only 3.0% of women had a known firstdegree relative with breast cancer, and 3.6% had a known second-degree family member.

### Pre-operative evaluation and surgical management

Table 2 shows the pre-operative evaluation was identified for 184 women and included a mammogram in 98.4% and ultrasound in 96.8% of women. A total of 180 women had undergone a breast operative intervention. A diagnostic core or incisional biopsy was identified for 44.4% of patients. An operative procedure performed with curative intent or local control was performed in 106 women and included a partial mastectomy (PM) in 21.7% (23/106) or a total mastectomy (TM) in 78.3% (83/106). Axillary dissection was performed in 40.1% (71/177) women. Patients with stage 1 to 3 disease were significantly more likely to have undergone an axillary dissection than patients with stage 4 disease (OR= 2.6 95% CI (1.7, 4.0, p<0.001).

**Table 2:** Surgical management of breast cancer patients

Variable	Number(%)
**Pre-operative evaluation (n=184)**	
**Mammogram**	181(98.4)
**Ultrasound**	179 (96.8)
**Surgical procedure**	
**Biopsy only (n=180)**	80 (44.4)
**Partial Mastectomy (n=106)**	23 (21.7)
**Mastectomy (n=106)**	83 (78.3)
**Axillary dissection (n=177)**	
**Yes**	71(40.1)
**No**	106 (59.9)
**Postoperative complication (n=183)**	
**Yes**	17 (9.3)
**No**	166 (90.7)
**Complication (n=17)**	
**Surgical site infection**	7 (41.1)
**Seroma, not infected**	4 (23.5)
**Seroma, infected**	1 (5.9)
**Profuse bleeding from a tumour**	1 (5.9)
**Swelling of breast**	1 (5.9)
**Surgical site bleeding**	1 (5.9)
**Wound ulceration**	1 (5.9)
**Wound dehiscence**	1 (5.9)
**Blood transfusion (n=181)**	
**Yes**	5 (2.8)
**No**	176 (97.2)

A postoperative complication was described for 17 of 183 women (9.3%), as shown in Table 2. The most common complication was a surgical site infection (3.8%). The mean length of postoperative hospital stay for all admissions was 8.6 ± 7.4 days (range 0 to 34 days).

A blood transfusion was administered to 5 (2.8%) women. Often the postoperative hospital stay included administration of chemotherapy.

However, there was no significant correlation between length of hospital stay and neoadjuvant chemotherapy administration.

### Non-surgical management

The frequency of all non-operative treatment modalities by stage is shown in Table 3. Neoadjuvant chemotherapy was administered to 64.5% of patients staged. Advanced stage 3 and 4 cancer patients were twofold more likely to receive neoadjuvant therapy than patients with earlier stage disease (OR= 2.0 95% CI (1.4, 3.0, p<0.001). Adjuvant radiation therapy was administered more often to earlier stage patients compared with those with advanced stages (OR= 54 95% CI (1.2, 24.7, p<0.031). There was a significant correlation between young age and administration of neoadjuvant radiation treatments (Pearson correlation r= -.175, p=0.02). There was no other significant association between chemotherapy, radiation therapy and oral anti-oestrogen medication administration and stage of the disease.

**Table 3:** Non-surgical breast cancer management

	Chemotherapy		Radiation Therapy		Anti-estrogen	
Stage	Neoadjuvant n(%)	Adjuvant n(%)	Neoadjuvant n(%)	Adjuvant n(%)	Tamoxifen n(%)	Anastrozole n(%)
**0**	0 (0.0)	0 (0.0)	0 (0.0)	0 (0.0)	1 (100.0)	1 (100.0)
**1**	0 (0.0)	1 (20.0)	0 (0.0)	1 (25.0)	2 (40.0)	0 (0.0)
**2**	16 (48.5)	11 (33.3)	1 (3.0)	1 (3.0)	1 (2.9)	0 (0.0)
**3**	52 (68.4)	35 (46.7)	0 (0.0)	1 (1.4)	11 (14.7)	4 (5.3)
**4**	50 (73.5)	13 (18.6)	2 (3.1)	0 (0.0)	7 (10.0)	2 (2.9)
**Total** *	118 (64.5)	60 (32.6)	3 (1.8)	3 (1.8)	22 (11.9)	7 (3.8)

* Total is expressed as total number and percent of patients who received the specific therapy from a cohort of 184 patients

### Histopathology

Histology was available for 164 patients. Invasive ductal carcinoma not otherwise specified (NOS) was the most pathological type representing 88.4%. Other pathological types included infiltrating lobular 16(9.8%), Paget's disease 2(1.2%) and intraductal carcinoma 1(0.6%). Further histology characteristics of breast cancer are presented in Table 4. More than four in every ten women were diagnosed with Grade 2 (43.8%) and Grade 3 (45.2%) breast cancers. There was a near even distribution comparing laterality of breast cancer, with 47.7% patients diagnosed with right breast cancer, 50.3% with left breast cancer, and 2.1% with bilateral cancer. The upper outer quadrant was the most common site of cancer (32.9%), while the nipple areola complex (0.7%) was the least common site of cancer.

**Table 4:** Histological characteristics of breast cancer

Characteristics	Number (%)
**Laterality of breast cancer (n=193)**	
**Right**	92 (47.7)
**Left**	97 (50.3)
**Bilateral**	4 (2.1)
**Quadrant of breast cancer (n=149)**	
**UOQ**	49 (32.9)
**UIQ**	17 (11.4)
**LOQ**	10 (6.7)
**LIQ**	14 (9.4)
**Retroareolar**	28 (18.8)
**Overlapping sites**	30 (20.1)
**Nipple areolar complex**	1 (0.7)
**Size of cancer (n=63)**	
**< 2.00 cm**	2 (3.2)
**2.00 to 4.99 cm**	27 (42.9)
**5.00 cm or greater**	34 (54.0)
**Grade of cancer (n=146)**	
**Grade 1**	16 (11.0)
**Grade 2**	64 (43.8)
**Grade 3**	66 (45.2)
**Lymphatic vascular invasion status (n=125)**	
**Present**	59 (47.2)
**Absent**	66 (52.8)
**Estrogen receptor status (n=95)**	
**Positive**	31 (32.6)
**Negative**	64 (67.4)
**Progesterone receptor status (n=96)**	
**Positive**	21 (21.9)
**Negative**	75 (78.1)
**Her-2/neu status (n=89)**	
**Positive**	29 (32.6)
**Negative**	60 (67.4)
**Triple Negative Cancers (n=89)**	
**TNBC**	41 (46.1)
**Not TNBC**	48 (53.9)
**Number of Axillary Lymph Nodes (n=87)**	
**None**	20 (23.0)
**1**	16 (18.4)
**2 to 4**	21 (24.1)
**5 to 10**	22 (25.3)
**> 10**	8 (9.2)

More than half (54%) of breast cancers reported were 5 centimetres (cm) or greater (mean 6.5+ 4.0 cm, range 1.5 - 20.0 cm). Estrogen receptor status was positive in 32.6%, and progesterone receptor status was positive in 22.1% of patients reported. Her-2/neu was positive in 32.6% of patients, while lymphatic vascular invasion (LVI) was identified in 47.2%. There was no significant correlation between age and histology, histologic grade or stage of the disease. There was no significant association between oestrogen receptor status, progesterone status, LVI and menopause status or age at diagnosis 50 years or younger.

However, Her-2/neu was significantly more likely to be positive in premenopausal women compared with a combined group of peri and post-menopausal women (52.4% versus 20.8% respectively, Odds ratio [OR] = 4.20, 95% CI 1.42–12.41, p=0.009).

There was no significant association between a positive Her-2/neu status and patients aged 50 years or younger. Decreasing age was significantly correlated with positive Her-2/neu cancers (Pearson r=.275, p<0.19). Tumour marker assessment was available for most patients who had an operative procedure performed.

Neither age nor menopause status was significantly associated with any other tumour marker. A combination of estrogen receptor, progesterone receptor, and Her-2/neu data were all available for 89 patients. Triple-negative breast cancer (TNBC) was identified for 41 (46.1%) patients. There was also no statistical significance between TNBC and age, stage of disease, menopause status, histology, size of cancer, the total number of positive axillary lymph nodes, and time to local recurrence, disease status, and death from breast cancer.

The results of the axillary dissection were available for 87 patients. Positive axillary lymph nodes, ranging from 1 to more than 10, were identified in 77.0 % of 87 patients. Approximately 10% of patients had more than ten positive nodes identified, as shown in Table 4.

No other sites of regional nodes (i.e. cervical, supraclavicular or infra-clavicular lymph nodes) or sites of distant disease (i.e. liver, lung) were reported to be biopsied.

### Treatment Outcomes

**Figure 2:**
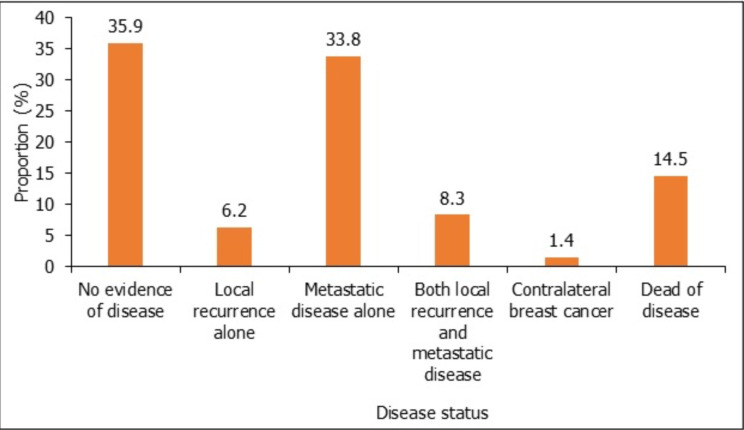
Disease status of breast cancer patients (n=145)

The mean duration of follow-up was 10.8 ± 9.8 months (range 0.3 to 49.31 months). Fifty-two (26.4%) of the initial cohort of 197 patients were reported to be lost to follow-up. Disease status follow up was available for 145 patients, as shown in Figure 2. A total of 21(14.5%) patients were reported to have succumbed to breast cancer. The mean duration from diagnosis to death was 4.6 ± 4.7 months (range 1 to 15 months). The mean duration from diagnosis to local recurrence after treatment for a curative intent was 13.2 ± 9.4 months (range 3 to 36). The mean time for diagnosis of metastatic disease was 14.0 ± 8.0 months (range 5 to 24). There were eight (5.5%) patients with metastatic disease to other sites, of which 5(62.5%) had metastasis to the liver while the other 3(37.5%) had metastasis to both the brain and lungs.

## Discussion

Breast cancer is a growing public health concern in Ghana that requires new and effective interventions based on population-specific characterization of the disease. Being the first reported study from a peripheral region in Ghana, the current study assesses breast cancer presentation, treatment, and outcomes among women in Cape Coast, Ghana. The mean age of women with breast cancer in this study was 49.9 ± 12.9 years. The majority of these women (54.8%) were 50 years old or younger, and 66% of women were peri-menopausal or post-menopausal at the time of diagnosis. For 84.3% of women, this was their primary presentation of breast cancer, suggesting that a proportion of these initially present with breast cancer and are lost to follow-up. Our finding on age fits the expected young profile of breast cancer patients in this region. It is comparable with prior studies in Accra (48.1years)^9^, Kumasi (49.1 years)^11^ and in Africa (50.2 years)^2^ in general. Apart from genetics, the young age of presentation could be explained by the youthful population structure of Ghana, as in other African countries. A majority (78.8%) of the patients in the study were identified with advanced disease (stage 3 or 4). Prior research has shown that Ghanaian women tend to present at a younger age, a more advanced stage (3 and 4), have larger tumours, and have fewer tumours that are hormone positive.^13^

Barriers in the diagnostic process and/or delays in seeking care could explain why women in this study may have presented with advanced stage of breast cancer. Some of the barriers in the diagnostic process may include the lack of current national breast cancer screening protocols or countrywide literacy initiatives, limited availability of mammography or ultrasound machines, or out-of-pocket expenses incurred with regards to receptor evaluation and definitive treatment.^13^ In most of the patients in this study, both a mammogram (98.4%) and an ultrasound (96.8%) was performed before the biopsy, suggesting that diagnostic imaging at the Cape Coast Teaching Hospital was accessible. This suggests that radiographic imaging may be available for screening purposes and should be incorporated into general health care recommendations for women.

Many women could not afford chemotherapy and would miss cycles of chemotherapy before returning with more advanced disease. It is also possible that some of these women presented with advanced diseases that made them poor candidates for other definitive treatments. There were instances where some of these patients rejected surgery after diagnostic biopsy either due to fear of losing breast or finances. This also explains why patients with advanced disease were more likely to undergo a diagnostic biopsy only in this study. The findings also showed that only 21.7% of the patients who had breast surgery underwent a partial mastectomy. Patients with Stage 1 to 3 cancer were more likely to undergo an axillary dissection than patients with Stage 4 disease, for whom treatment options were limited to palliative options.

There are multiple reasons why women present with advanced stages of disease. Clegg-Lamptey found that some were due to delays caused by previous medical consultations or ignorance or the painless nature of the lump and the fact that the patients thought the lump might disappear^9^ Further, women in Ghana are likely to resort to traditional or spiritual treatment methods, and when those methods fail, they turn to orthodox medical advice.^16^ This data signifies that more public education is needed and an expansion of the benefit package of the National Health Insurance Scheme (NHIS) to cover the costs associated with the diagnostic process and lessen the possibility of defaulting from care.

Cape Coast Teaching Hospital offers chemotherapy onsite, which enhances the ability of patients to access this form of treatment. This was evident in the data showing that 64.5% of patients that were staged received neoadjuvant chemotherapy. Patients with advanced stage breast cancer were also more likely to receive neoadjuvant therapy than patients presenting with earlier stage disease. They were often too advanced to obtain other types of definitive treatments. The current study revealed that adjuvant radiation therapy was administered more often to patients with earlier stages of breast cancer.

The definitive care for patients with early-stage tumours was a partial mastectomy in addition to adjuvant radiation therapy post-surgery. None of the patients with advance or metastatic disease received adjuvant radiotherapy. Several plausible reasons could account for this, including patients' unwillingness to patronize the radiotherapy services due to cost, geographic distance for radiotherapy services or hopelessness associated with the advanced nature of the disease. Indeed, adjuvant radiation therapy was more difficult to access because it was not offered at Cape Coast Teaching Hospital. The closest hospital that had radiation therapy was Korle Bu Teaching Hospital, located approximately 142.6 kilometres in Accra, Ghana. Perhaps at Cape Coast Teaching Hospital, it would be advantageous to create access for radiation therapy either on-site or by providing support for all patients to access radiation therapy at hospitals with that capacity. Other treatments such as tamoxifen and trastuzumab are not available at CCTH, perhaps because the National Health Insurance Scheme does not cover them. There are, however, a few local pharmacies from which they may be purchased but are unaffordable to most patients.

Breast cancer presents differently in African and African-American women compared to other populations.^6^,^14^ In the United States, black females have a lower overall incidence of breast cancer than white females, but are diagnosed with later-stage disease, have shorter survival, and have the highest mortality from breast cancer of all ethnic groups in the USA.^14^ In this study, only 3% of women had a known first-degree family history of breast cancer, and 3.6% had a known second-degree family member with breast cancer. Pathology data was limited perhaps because such services were not available at CCTH until recently, or patients were simply deterred by their already advanced stage of disease and associated costs. Consistent with previous studies in Ghana^8^–^11^, invasive ductal carcinoma was the most common breast cancer malignancy, with a majority of malignancies being either Grade 2 (43.8%) or Grade 3 (45.2%). This study also found that the most common cancer site was the upper outer quadrant, with 54% of cancers reported as 5 centimetres or greater.

Parkin et al. found that “there is little to no evidence for differences in histopathological type, but tumours in black females are of a higher grade, are more often hormone-receptor negative, and more often show characteristics of inflammatory breast cancer than those in white females” .^15^ Receptor status data was available for a fraction of the patients, as it is considered an out-of-pocket expense for patients. The present study found positive estrogen and progesterone receptors in 32.6% and 21.9% of patients.

The hormone receptor pattern in breast cancer patients is consistent with estrogen (47.1%) and progesterone (13.2%) receptors in Kumasi.^11^ A lower proportion (18%) of patients tested positive for estrogen receptor and/ or progesterone receptor from a similar study in Accra^8^ The difference in receptor pattern could be due to differences in methodologies used in the various studies, especially considering the minimal variations between the three cities (Accra, Kumasi and Cape Coast) in terms of the influence of demography and socioeconomic status. In an international study, the proportions with TBNC disease were 82%, 26%, and 16%, respectively, among Ghanaian, African American, and white American^6^ in the present study, less than half (46.1%) of patients were diagnosed with TBNC. Oestrogen receptor and progesterone receptor-positive breast cancers are typically associated with higher survival rates than triple-negative breast cancer. Other factors that are associated with poor survival include lymphatic vascular invasion and axillary node involvement. This study found that lymphatic vascular invasion was present in 47.2% of tumours.

Ideally, at least ten nodes should be dissected and examined for optimal prognostic staging, 16,17, but in the present study, one-fourth (25.3%) of patients had 5 to 10 nodes examined while only 9.2% of them had at least ten nodes examined. Although the number of nodes removed per patient was lower than the required, positive axillary lymph nodes were found in 77.0% of the 87 patients (OR= 2.6 95% CI (1.7, 4.0, p<0.001) who underwent an axillary dissection with nodal status recorded.

The data may be an underestimation in patient outcome as efforts to follow-up on over a quarter of patients following initial clinic visits were unsuccessful due to inaccuracies in contact information (phone numbers and addresses). Although similar studies in Ghana did not report mortalities due to breast cancer 8–11, our study estimated a case fatality rate of 14.5% from breast cancer mainly due to the advanced nature of the disease. Compared to a previous study in Accra^9^ which found 22.2% (35/158) of patients with metastatic disease, we documented that 5.5% of our patients in Cape Coast had metastatic disease mainly in the liver. The proportion of patients with metastatic disease could be attributed to discrepancies in data sources and lost to follow-ups. Such patients often deteriorated so fast that the mean duration from the time of their diagnosis to death was 4.6 +/- 4.7 months, with over six in ten (62.9%) of them dying within one month from initial presentation to the hospital. Such poor outcome underscores the need for more innovative measures to ensure early detection for timely remedy interventions.

Much as the study provides some important additional insights into the breast cancer situation in Ghana, some limitations need to be considered in interpreting the findings. Firstly, the study was based on hospital records, which could underestimate the actual number of breast cancer patients within Cape Coast and surrounding areas. Secondly, as typically the case with most retrospective studies of this nature, poor record-keeping meant that several patients had to be left out of the sample due to inaccuracies and missing information.

## Conclusion

Breast cancer patients in CCTH typically present at advanced stages with poor prognosis and outcome due to a myriad of social, cultural, economic, and logistical constraints. This calls for interventional efforts to promote early care seeking behaviours among women with breast related conditions.
